# Sex-stratified and ascorbic acid intake-modified associations between body roundness index and biological aging: a NHANES-based study on interactions and mediation

**DOI:** 10.1186/s12944-025-02708-1

**Published:** 2025-09-19

**Authors:** Xinxing Wang, Xiaoxiao Qu, Guosong Jiang

**Affiliations:** 1 Department of Clinical Laboratory, Chengdu BOE Hospital, Chengdu, 610000 Sichuan China; 2 Department of Clinical Laboratory, The Second Affiliated Hospital & Yuying Children’s Hospital of Wenzhou Medical University, Wenzhou, 325000 Zhejiang China; 3 Department of Pulmonary and Critical Care Medicine, The Frist People’s Hospital of Zhaotong &Zhaotong Hospital Affiliated to Kunming Medical University, Zhaotong, 657000 Yunnan China

**Keywords:** Biological aging, Body roundness index, Ascorbic acid, Sex differences, Interaction analysis, Mediation analysis

## Abstract

**Background:**

Biological aging, defined as the biological age (BA) or phenotypic age (PA) exceeding the chronological age (CA), is a key indicator of premature aging. Obesity accelerates aging; however, the effect of Body Roundness Index (BRI), an indicator of abdominal obesity, combined with sex or Ascorbic acid (Asc), on biological aging remains unclear. This study examined the association between BRI and biological aging, its interaction with sex and Asc intake, and its mediating mechanisms.

**Methods:**

Data from the 1999–2018 U.S. National Health and Nutrition Examination Survey (NHANES) formed the basis of this study. Biological aging is characterized by BA or PA surpassing CA. Association between the BRI and biological aging was evaluated using multivariable-adjusted weighted regression. To assess for nonlinearity, restricted cubic splines were utilized, while interactions were investigated through both additive and multiplicative analyses. The bootstrap method was used to examine the potential mediating effects of biomarkers of metabolic dysfunction, oxidative stress, and protective pathways.

**Results:**

The final analysis included 14,337 U.S. adults (mean age 47.5 years; 50.27% women). A total of 49.32% of the participants showed signs of biological aging, with BRI being positively associated with biological aging in a nonlinear manner, including a threshold effect. The increase in the risk per BRI unit was more significant in women, and high doses of Asc reduced the risk of biological aging associated with increased BRI. Mediation analysis indicated that the association between BRI and accelerated aging was partly mediated by metabolic dysfunction (mediated by the triglyceride-glucose index [18.73%] and by triglycerides [9.21%], oxidative stress mediated by uric acid [17.92%] and by white blood cell count [5.80%], and depletion of protective factors mediated by vitamin D [− 3.85%] and by high-density lipoprotein [− 4.24%]). A sensitivity analysis confirmed the reliability of the findings.

**Conclusions:**

A higher BRI showed a nonlinear positive association with accelerated biological aging, a relationship that appears to be modified by female sex and Asc intake. Clinical approaches targeting metabolic dysfunction, oxidative stress, and antioxidant and vasoprotective reserves may help combat obesity-related aging. BRI thresholds enable early identification of individuals at high risk for personalized interventions.

**Graphical abstract:**

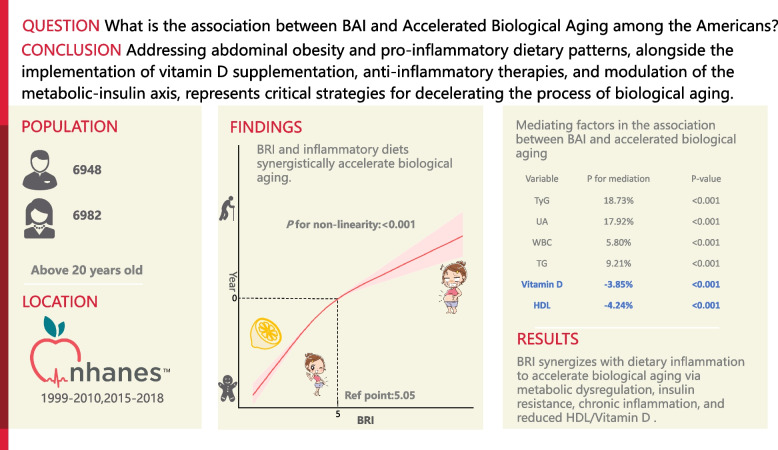

**Supplementary Information:**

The online version contains supplementary material available at 10.1186/s12944-025-02708-1.

## Research Insights

**Table:** 

What is currently known about this topic?
1. Obesity, particularly abdominal obesity with visceral fat accumulation, accelerates biological aging through metabolic dysfunction and oxidative stress
2. Sex differences exist in fat distribution and aging susceptibility, yet the modifying role of dietary antioxidants (e.g., Asc) remains underexplored
3. Traditional metrics such as BMI inadequately reflect abdominal obesity, whereas the BRI has been established as a superior predictor of disease onset and prognosis
What is the key research question?
This study investigates whether the BRI, a visceral adiposity metric, exhibits a nonlinear association with biological aging, how sex and Asc intake independently modify this relationship, and through which metabolic and oxidative pathways these effects are mediated
What is new?
1. We identified a nonlinear positive association with threshold effects between BRI and biological aging
2. Independent modifiers were revealed: females exhibit heightened vulnerability to BRI-associated aging, while high Asc intake demonstrates protective effects, with additive and multiplicative interactions observed
3. Comprehensive mediation framework: metabolic dysfunction (TyG, TG), oxidative stress (UA, WBC), and depletion of protective factors (vitamin D, HDL-C) collectively explain 41.6% of BRI’s aging effect
How might this study influence clinical practice?
BRI thresholds enable early identification of high-risk individuals for targeted monitoring. Asc-rich dietary guidelines may mitigate aging risks, particularly in females with central obesity. Sex-stratified interventions targeting metabolic-oxidative pathways (e.g., TyG reduction, vitamin D/HDL preservation) could optimize anti-aging strategies

## Background

Biological aging, defined as the condition in which the biological age (BA) or phenotypic age (PA) exceeds the chronological age (CA) [1, 2], is a key indicator of premature aging and is associated with increased mortality risk and age-related functional decline [3–5]. Research indicates that 19.7–47.6% of American adults show signs of biological aging [6], with obesity and dietary intake emerging as modifiable influencing factors—modifiable drivers linked to aging via metabolic dysregulation and oxidative stress [7–10]. Despite critical findings, it remains unclear whether antioxidant intake, such as Ascorbic acid (Asc) supplementation and sex differences independently interact with abdominal obesity (quantified by the Body Roundness Index [BRI]) to modify biological aging trajectories. Further investigation is needed to identify the biological pathways underlying these potential interactions for developing targeted interventions aimed at modulating aging processes.

Recent evidence underscores the significance of visceral adiposity and antioxidant micronutrient status as chief determinants of biological aging [7–10]. Abdominal obesity, defined as excess visceral fat, facilitates insulin resistance (IR), chronic inflammation, and oxidative stress, which are fundamental mechanisms of acceleration of the biological aging process [6, 8]. Consequently, the intake of low-antioxidant foods may exacerbate these pathways and potentially intensify obesity-related aging. Importantly, sex differences in fat distribution suggest that women may be more susceptible to accelerated biological aging associated with adiposity [11], although this hypothesis is yet to be empirically tested. The BRI, a metric derived from waist circumference that accurately quantifies visceral adiposity, presents a unique opportunity to explore these relationships [12]. Unlike the body mass index (BMI), the BRI effectively captures the central fat distribution and addresses the limitations of BMI in distinguishing between lean mass and adiposity [13], serving as a reliable predictor of the prevalence of various diseases, prognosis, and overall mortality within the population [13–15]. However, their association with dual biological aging metrics (BA and PA), interactions with Asc intake, and sex differences remain unexplored. Therefore, this study was designed to clarify the complex relationship between the BRI and biological aging. The study aimed to address: i) the precise nature of the association between the BRI and biological aging (whether linear or nonlinear), ii) the influence of sex and Asc intake on this relation, and iii) the potential biological processes (metabolic dysfunction, inflammation, and protective factors) involved.

To address this gap, a three-phase analysis was conducted using data from the National Health and Nutrition Examination Survey (NHANES). First, the association between the BRI and dual biological age metrics (BA and PA) and their nonlinear characteristics was investigated. Second, the interaction effects of key modifiers, specifically Asc intake and sex, on the relationship between BRI and biological aging were analyzed. Finally, the mechanisms underlying these associations were explored. The goal of synthesizing these findings is to develop a comprehensive framework that could potentially enhance clinical risk stratification and help identify modifiable targets, such as dietary Asc intake and metabolic dysregulation, for interventions aimed at mitigating the acceleration of aging associated with obesity.

## Methods

### Data source

NHANES is a key research program of the National Center for Health Statistics (NCHS). It employs a stratified, multistage probability sampling methodology to assess the health and nutritional status of a non-institutionalized U.S. population through interviews, physical examinations, and laboratory tests. The study protocol was approved by the NCHS Research Ethics Review Board, and all participants provided written informed consent following the principles of the STROBE guidelines [16]. Comprehensive information on NHANES data can be found at https://www.cdc.gov/nchs/nhanes.

### Study population

**Fig. 1:**
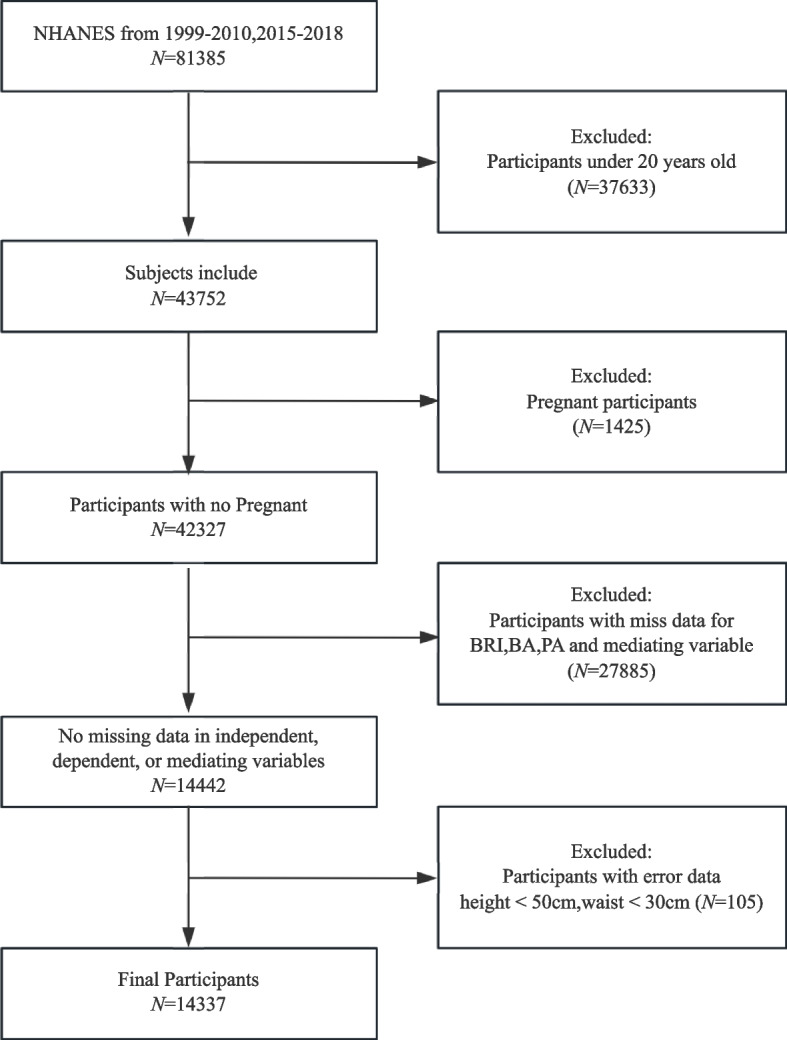
Participant inclusion flowchart (NAHNES)

Data from the 1999–2010 and 2015–2018 cycles of the NHANES were used for this analysis as these specific periods contained comprehensive datasets required for calculating both BA and PA. The final sample comprised 14,337 participants, while 67,048 individuals were excluded based on the following criteria: (1) age under 20 years, as questions about cardiovascular disease (CVD) and diabetes history were only posed to participants aged 20 years and above (*N* = 37,633); (2) pregnancy (*N* = 1,425); (3) missing parameters essential for the computation of BA, PA, and mediating variables (*N* = 27,885); and (4) extreme outliers with a height of < 50 cm or a waist circumference of < 30 cm (*N* = 105). Missing covariate data were analyzed using multiple imputations. Subsequently, BRI quartiles were used to divide the participants into four distinct groups following the data processing workflow illustrated in Fig. 1**.**

### Exposures

BRI was calculated as $364.2-365.5\times \sqrt{{1-\frac{\text{waist circumference}({\varvec{m}})}{2{\varvec{\pi}}}}^{2}/{\frac{\text{height}({\varvec{m}})}{2}}^{2}}$ [12]

In the NHANES, participants'height was assessed to the nearest 0.1 cm utilizing a stadiometer while they were barefoot. Waist circumference was determined at the midpoint between the lower rib margin and the iliac crest using a non-stretchable tape, with measurements recorded to the nearest 0.1 cm [16].

### Outcome

Two separate methodologies were used to assess biological aging, BA and PA. BA was computed using the Klemera and Doubal method, which integrates eight metabolic and inflammatory biomarkers, including ln-C-reactive protein (ln-CRP) and systolic blood pressure [17, 18]. In contrast, PA was derived from a multivariate mortality risk model incorporating ten biomarkers reflecting systemic health, such as albumin level, lymphocyte percentage, and red cell distribution width [19]. An individual was classified as experiencing biological aging if their BA or PA was greater than their age (CA), creating a binary outcome variable [1]. The specific formulae used for these calculations are detailed in Supplementary Material 1.

### Covariables

Based on published research findings related to biological aging, clinical factors, and dietary associations [6, 20–23], this study incorporated several potential confounding variables, including age, sex, race, marital status, education level, poverty income ratio (PIR), physical activity, smoking and drinking status, CVD, hypertension, diabetes mellitus (DM), dietary fiber intake, dietary inflammation, dietary zinc intake, and Asc intake. The detailed definitions of the variables are provided in Supplementary Material 2.

### Mediating variable

Several potential mediators were examined in this study, with the triglyceride-glucose (TyG) index being a notable example, utilized as a surrogate for IR; uric acid (UA) and triglyceride (TG), indicative of metabolic abnormalities; white blood cell (WBC) count, reflecting inflammatory status; vitamin D (VITD), representing antioxidant capacity; and high-density lipoprotein (HDL), to evaluate vascular protection. The TyG index was computed using the following formula: TyG = ln [TG (mg/dL) × glucose (mg/dL)/2] [24].

### Statistical analysis

Considering the complex, multi-stage cluster sampling methodology of the NHANES, sampling weights were applied following the guidelines provided on the official NHANES website to ensure national representativeness of the data. Specifically, the sample weight code"WTMEC4YR"was utilized for the 1999–2002 period, whereas"WTMEC2YR"was employed for the 2003–2010 and 2015–2018 periods. The sampling weights were calculated as follows: for the 1999–2002 cycle, the weight was determined as 2/8 × WTMEC4YR, and for the subsequent cycles, it was calculated as 1/8 × WTMEC2YR. Multiple imputations (five iterations) were used to address the missing covariate values. Supplemental Table 1 provides a detailed account of the missing values. The baseline characteristics of the study cohort were stratified according to the BRI quartiles. Continuous variables are reported as means with standard errors (SE), while categorical data are presented as counts and percentages (%). To compare groups, the Wilcoxon rank-sum test was applied to continuous data, accommodating a complex survey design. Categorical data were analyzed using the chi-square test adjusted with Rao and Scott's second-order correction.

**Table 1:** Baseline characteristics of the study participants stratified by BRI quartile

Characteristic	Total	Q1 (≤ 3.83)	Q2 (3.84–5.06)	Q3 (5.06–6.60)	Q4 (≥ 6.60)	*P-*value ^a^
Weighted Population, *N*	*N* = 77,297,419	*N* = 22,176,572	*N* = 19,783,137	*N* = 18,000,902	*N* = 17,336,809	
Unweighted Population, *N*	*N* = 14,337	*N* = 3584	*N* = 3584	*N* = 3584	*N* = 3585	
**Demographic**						
Age, mean (SE), y	47.50 (0.27)	40.34 (0.38)	48.02 (0.35)	51.29 (0.38)	52.12 (0.42)	< 0.001
Sex, n (%)						< 0.001
Male	7,211 (49.73%)	1,914 (49.44%)	2,045 (56.05%)	1,869 (53.14%)	1,383 (39.35%)	
Female	7,126 (50.27%)	1,670 (50.56%)	1,539 (43.95%)	1,715 (46.86%)	2,202 (60.65%)	
Race, n (%)						< 0.001
Non-Hispanic White	6,672 (70.14%)	1,777 (71.50%)	1,671 (70.32%)	1,590 (68.92%)	1,634 (69.45%)	
Non-Hispanic Black	2,775 (10.28%)	785 (10.92%)	603 (8.64%)	621 (9.29%)	766 (12.38%)	
Mexican American	2,578 (7.98%)	396 (5.20%)	656 (8.21%)	809 (10.15%)	717 (9.04%)	
Other Hispanic	1,174 (4.88%)	223 (4.31%)	310 (5.07%)	311 (5.22%)	330 (5.03%)	
Other ^b^	1,138 (6.72%)	403 (8.07%)	344 (7.77%)	253 (6.43%)	138 (4.10%)	
Marital status, n (%)	8,799 (65.14%)	2,006 (60.03%)	2,370 (69.22%)	2,348 (69.15%)	2,075 (62.86%)	< 0.001
PIR ^c^, n (%)						< 0.001
< 1.30	3,700 (18.86%)	832 (17.18%)	856 (17.17%)	947 (19.35%)	1,065 (22.47%)	
≥ 1.30–3.50	5,273 (37.09%)	1,288 (36.42%)	1,275 (35.09%)	1,346 (37.90%)	1,364 (39.39%)	
> 3.50	4,209 (44.05%)	1,192 (46.40%)	1,167 (47.73%)	1,010 (42.75%)	840 (38.14%)	
Education level, n (%)						< 0.001
Less than 9th grade	1,712 (5.86%)	220 (3.52%)	414 (5.41%)	557 (7.78%)	521 (7.39%)	
9-11th Grade	2,065 (10.54%)	493 (9.83%)	481 (9.35%)	493 (10.81%)	598 (12.51%)	
High school or equivalent	3,416 (25.21%)	831 (23.01%)	811 (24.20%)	908 (27.49%)	866 (26.79%)	
Some College or AA degree	4,073 (30.67%)	1,047 (29.72%)	1,002 (30.65%)	966 (29.79%)	1,058 (32.84%)	
College graduate or above	3,059 (27.72%)	990 (33.92%)	871 (30.39%)	657 (24.13%)	541 (20.47%)	
**Lifestyle**						
Smoking status, n (%)						< 0.001
Never ^d^	7,544 (52.03%)	1,877 (52.44%)	1,931 (53.03%)	1,881 (51.51%)	1,855 (50.93%)	
Former ^e^	3,710 (26.17%)	674 (20.25%)	892 (25.02%)	1,070 (30.29%)	1,074 (30.79%)	
Current ^f^	3,073 (21.79%)	1,030 (27.31%)	760 (21.95%)	630 (18.20%)	653 (18.29%)	
Alcohol intake, n(%)						< 0.001
Never ^g^	1,784 (10.93%)	362 (9.54%)	415 (9.78%)	485 (12.00%)	522 (12.95%)	
Former ^h^	2,284 (14.12%)	387 (9.41%)	502 (12.45%)	650 (16.26%)	745 (19.98%)	
Current ^i^	9,121 (74.96%)	2,572 (81.06%)	2,401 (77.77%)	2,157 (71.74%)	1,991 (67.08%)	
Physical activity, mean (SE), min/week	166.25 (15.75, 645.00)	210.00 (35.10, 740.00)	189.00 (30.00, 709.85)	144.05 (10.00, 600.00)	120.00 (0.00, 480.00)	< 0.001
**Health Status**						
CVD ^j^, n (%)	1,646 (8.71%)	188 (3.90%)	329 (7.11%)	478 (10.84%)	651 (14.47%)	< 0.001
Hypertension, n (%)	6,159 (37.40%)	783 (17.95%)	1,330 (33.32%)	1,827 (45.55%)	2,219 (58.47%)	< 0.001
DM, n (%)	2,807 (14.41%)	195 (3.78%)	506 (9.51%)	795 (17.00%)	1,311 (30.89%)	< 0.001
**Dietary Intake**						
Dietary fiber, mean (SE), gm	14.40 (9.70, 20.70)	14.90 (10.10, 21.20)	14.88 (9.90, 21.20)	14.40 (9.50, 20.70)	13.70 (9.10, 19.60)	< 0.001
DII, mean (SE)	1.73 (0.22, 2.94)	1.49 (−0.03, 2.75)	1.58 (0.05, 2.78)	1.86 (0.37, 3.04)	2.10 (0.56, 3.17)	< 0.001
Zinc intake, mean (SE), mg	10.33 (7.03, 14.88)	10.53 (7.19, 15.32)	10.59 (7.35, 15.27)	10.40 (7.00, 14.73)	9.68 (6.59, 14.09)	< 0.001
Ascorbic Acid intake, mean (SE), mg	51.10 (21.80, 110.80)	55.70 (23.30, 117.68)	54.29 (22.83, 116.12)	47.00 (20.60, 110.10)	46.53 (20.20, 93.70)	< 0.001
**Anthropometric Measures**						
TG, mean (SE), mg/dl	106.00 (73.00, 156.00)	80.00 (58.00, 111.00)	107.00 (74.00, 156.00)	123.00 (86.00, 177.00)	129.00 (91.00, 184.00)	< 0.001
WBC, mean (SE),10⁹ cells/L	6.79 (0.03)	6.30 (0.05)	6.65 (0.05)	6.96 (0.05)	7.41 (0.05)	< 0.001
VITD, mean (SE), nmol/L	67.14 (0.64)	70.84 (0.80)	69.41 (0.83)	65.40 (0.74)	61.64 (1.01)	< 0.001
UA, mean (SE), mg/dl	5.48 (0.02)	4.95 (0.03)	5.42 (0.03)	5.71 (0.03)	6.00 (0.03)	< 0.001
HDL, mean (SE), mg/dl	54.11 (0.23)	61.03 (0.38)	54.16 (0.37)	50.70 (0.36)	48.74 (0.31)	< 0.001
TyG, mean (SE)	8.63 (0.01)	8.27 (0.01)	8.63 (0.01)	8.81 (0.01)	8.90 (0.02)	< 0.001
**Biological aging conditions**						
Biological Age, mean (SE), y	47.63 (0.26)	39.47 (0.36)	47.77 (0.33)	51.71 (0.37)	53.68 (0.37)	< 0.001
Biological Aging ^k^, n (%)						< 0.001
No	7,229 (50.68%)	2,112 (60.97%)	1,966 (54.24%)	1,750 (47.45%)	1,401 (36.82%)	
Yes	7,108 (49.32%)	1,472 (39.03%)	1,618 (45.76%)	1,834 (52.55%)	2,184 (63.18%)	
Phenotypic age, mean (SE), y	45.30 (0.30)	35.29 (0.42)	44.68 (0.38)	49.70 (0.42)	54.23 (0.43)	< 0.001
Biological Aging ^l^, n (%)						< 0.001
No	9,320 (68.11%)	2,822 (81.91%)	2,572 (74.49%)	2,279 (65.27%)	1,647 (46.15%)	
Yes	5,017 (31.89%)	762 (18.09%)	1,012 (25.51%)	1,305 (34.73%)	1,938 (53.85%)	

All means and SEs for continuous variables and percentages for categorical variables were weighted

*BRI* Body Roundness Index, *PIR* Poverty income ratio, *CVD* Cardiovascular disease, *DM* Diabetes mellitus, *DII* Dietary Inflammatory Index, *TG* Triglyceride, *WBC* White blood cell, *VITD* Vitamin D, *UA* Uric acid, *HDL* High-Density Lipoprotein, *TyG* Triglyceride-glucose index, *SE* Standard Error

^a^ Wilcoxon rank-sum test for complex survey samples; chi-squared test with Rao & Scott's second-order correction

^b^ Including Multi-Racial

^c^ Categorized into the following 3 levels based on the family poverty income ratio: < 1.3, ≥ 1.3 to 3.5, and > 3.5

^d^ Defined as individuals who have smoked fewer than 100 cigarettes in their lifetime

^e^ Defined as individuals who have smoked more than 100 cigarettes in their lifetime and do not smoke at all now

^f^ Defined as individuals who have smoked more than 100 cigarettes in their lifetime and smoke some days or every day

^g^ Defined as individuals who have had fewer than 12 drinks in their lifetime

^h^ Defined as individuals who have had 12 or more drinks in a year but did not drink in the past year, or who did not drink in the past year but have had 12 or more drinks at some point in their lifetime

^I^ Current drinking status includes: light drinking (≤ 1 drink per day for women, ≤ 2 drinks per day for men); moderate drinking (≥ 2 drinks per day for women, ≥ 3 drinks per day for men, or binge drinking ≥ 2 days per month); heavy drinking (≥ 3 drinks per day for women, ≥ 4 drinks per day for men, or binge drinking on ≥ 5 days per month [≥ 4 drinks per occasion for women, ≥ 5 drinks per occasion for men])

^j^ Defined as: A self-reported prior diagnosis of coronary heart disease, angina pectoris, stroke, myocardial infarction, or congestive heart failure, with the presence of any one of these conditions constituting a confirmed diagnosis

^k^ Biological aging as defined by Biological Age

^l^ Biological aging as defined by Phenotypic age

A multivariate weighted logistic regression model was used to investigate the association between the BRI and biological aging. Concurrently, a multivariate weighted linear regression analysis was conducted to evaluate the relationship between BRI and BA. Model 1 served as the unadjusted model and lacked covariate adjustment. Model 2 included adjustments for fundamental demographic characteristics of the study population, such as age, sex, and race. Model 3 was expanded by incorporating additional demographic and Lifestyle variables, including marital status, income, educational attainment, smoking status, alcohol consumption, and physical activity levels. Model 4 introduced further adjustments for chronic diseases and dietary characteristics, including hypertension, diabetes, CVD, dietary fiber intake, dietary inflammatory index, and Asc and Zinc intake. A linear regression model was rigorously assessed to ensure compliance with all fundamental assumptions, including posterior predictive checks, linearity, homoscedasticity, influential observations, collinearity, and the normality of residuals. Similarly, all logistic regression models were evaluated to confirm their adherence to these criteria, including posterior predictive checks, binned residuals, influential observations, and collinearity (Supplemental Figs. 1 and 2).

To investigate the potential nonlinear dose–response relationship between BRI and BA, as well as its rate of change, this study utilized a restricted cubic spline model. BRI was modeled as a continuous predictor utilizing restricted cubic splines with four knots placed at the 5th, 35th, 65th, and 95th percentiles. All spline models were adjusted for the set of covariates in Model 4. Following the analysis of the smoothed curve, a two-piecewise linear and logistic regression model was developed to detect threshold effects, incorporating adjustments for potential confounding variables.

To elucidate the potential interaction factors among diet, sex, and BRI, subgroup analyses were conducted following adjustments for model 4. These analyses were stratified according to dietary fiber intake, dietary inflammatory index, Asc and zinc intake, and sex. To prevent the introduction of category boundaries, we implemented a binary quantile approach for the continuous variables. For subgroups exhibiting a *P*-value for interaction of < 0.05, both additive and multiplicative interaction analyses were performed. To evaluate interaction effects, a likelihood ratio test was employed for multiplicative interactions. For potential additive interactions, three specific indicators were calculated: the relative excess risk due to interaction (RERI), the attributable proportion of interaction (AP), and the synergy index (SI) [25]. The absence of an additive interaction is signified by the confidence intervals for RERI and AP, which include 0, and the confidence interval for SI, which includes 1.

We performed a mediation analysis to investigate the mechanisms by which the BRI affects biological aging. To evaluate the mediating effects of TG, WBC, TyG, UA, VITD, and HDL on the relationship between the BRI and biological aging, a bootstrap resampling method with 1000 iterations was used.

Three sensitivity analyses were conducted to ensure the robustness of the findings. First, to mitigate bias from extreme values, the BRI distribution was trimmed at the 0.5th and 99.5th percentiles before modeling. Second, the association between the BRI and PA, as well as its acceleration, was examined. Third, we conducted a multivariate analysis of the BRI and BA risk by directly deleting missing covariates.

Statistical analyses were performed using the R Statistical Software (v4.2.2; R Foundation for Statistical Computing; http://www.R-project.org) and the Free Statistics analysis platform (v2.1.1, Beijing Free Clinical Medical Technology Co., Ltd.). Descriptive statistics were calculated for all participants, and statistical significance was assessed using a two-tailed test with a *P* value threshold of < 0.05.

## Results

### Characteristics of the population according to the BRI

This study included 14,337 participants, with weighted results extrapolated to represent the demographic characteristics of 77,297,419 adults in the United States, 50.27% of whom were females. The weighted mean age was 47.63 years (SE = 0.26) for the BA and 45.30 years (SE = 0.3) for the PA group. When biological aging was assessed using BA, 49.32% of the participants were identified as experiencing accelerated aging, compared with 31.89% when assessed using PA. Upon categorizing BRI into four distinct groups, older adults, women, Mexican Americans, low-income individuals, former smokers, drinkers, and those engaging in shorter durations of weekly physical activity exhibited elevated BRI. Additionally, elevated BRI was prevalent among individuals diagnosed with CVD, hypertension, and DM. Furthermore, participants with a lower intake of dietary fiber, zinc, and Asc demonstrated a higher BRI. Blood analysis of the high-BRI group revealed elevated levels of TG, WBC, UA, and TyG, along with reduced levels of VITD and HDL (Table 1).

### Association between BRI and biological aging

**Fig. 2:**
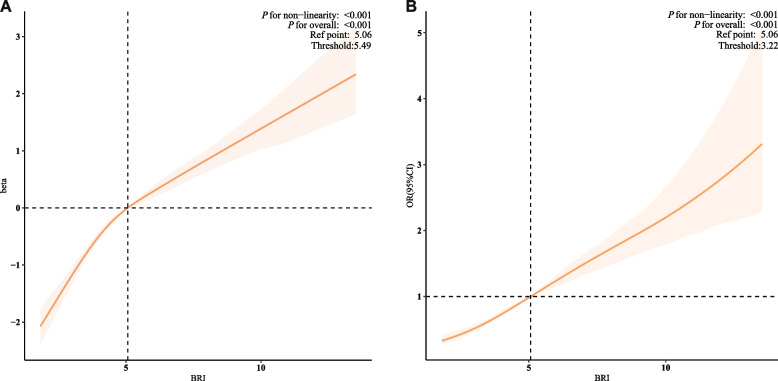
The dose–response associations of BRI with biological age (A), and the risk of biological aging (B) in US adults BRI: Body Roundness Index. These panels depict the dose–response relationships from weighted restricted cubic spline models, adjusted for covariates in Model 4. The solid orange Lines represent the estimated effects, and the shaded areas denote the 95% confidence intervals. Panel A shows the association between BRI and continuous biological age (BA). The horizontal dashed line indicates a null effect (β = 0), corresponding to the reference point at BRI = 5.06. The vertical dashed line at BRI = 5.49 indicates the threshold, which was statistically determined as the inflection point using a two-piecewise linear regression model. Panel B shows the association between BRI and the risk of biological aging (binary outcome). The horizontal dashed line indicates a null effect (Odds Ratio [OR] = 1), corresponding to the reference point at BRI = 5.06. The vertical dashed line at BRI = 3.22 indicates the threshold, identified using a two-piecewise logistic regression model. For both panels, the overall association and the nonlinear relationship are statistically significant (*P *for overall < 0.001, *P* for nonlinearity < 0.001)

As demonstrated in Table 2, following the adjustment for confounding variables in Model 4, each incremental unit increase in the BRI was correlated with a 0.37-year augmentation in BA (β = 0.37, 95% confidence interval [CI]: 0.33–0.42, *P* < 0.001) and a 25% elevated risk of biological aging (odds ratio [OR] = 1.25, 95% CI: 1.22–1.28, *P* < 0.001). In comparison to the group with the lowest BRI, the group with the highest BRI exhibited a 2.11-year increase in BA (β = 2.11, 95% CI: 1.86–2.35, *P* < 0.001) and a 3.52-fold elevated risk of biological aging (OR = 3.52, 95% CI: 2.74–3.69, *P* < 0.001). Furthermore, the analysis identified a nonlinear positive association between the BRI and biological aging, with inflection points observed at 5.49 for BA and 3.22 for biological aging (Fig. 2).

**Table 2:** Baseline characteristics of the study participants stratified by BRI quartile

	Categories	**Model 1**		**Model 2**		**Model 3**		**Model 4**	
		β(95%CI)	*P-*value	β(95%CI)	*P-*value	β(95%CI)	*P-*value	β(95%CI)	*P-*value
**biological age**	BRI	2.16 (1.97, 2.34)	< 0.001	0.59 (0.54, 0.63)	< 0.001	0.58 (0.53, 0.62)	< 0.001	0.37 (0.33, 0.42)	< 0.001
	BRI Quartile								
	Q1	0(Ref)		0(Ref)		0(Ref)		0(Ref)	
	Q2	8.30 (7.53, 9.07)	< 0.001	1.09 (0.87, 1.31)	< 0.001	1.10 (0.87, 1.32)	< 0.001	0.87 (0.64, 1.09)	< 0.001
	Q3	12.24 (11.30, 13.18)	< 0.001	2.01 (1.78, 2.25)	< 0.001	1.97 (1.73, 2.20)	< 0.001	1.40 (1.17, 1.63)	< 0.001
	Q4	14.21 (13.26, 15.16)	< 0.001	3.35 (3.10, 3.61)	< 0.001	3.31 (3.06, 3.55)	< 0.001	2.11 (1.86, 2.35)	< 0.001
	*P* for trend		< 0.001		< 0.001		< 0.001		< 0.001
**biological aging**		OR (95%CI)	*P-*value	OR (95%CI)	*P-*value	OR (95%CI)	*P-*value	OR (95%CI)	*P-*value
	BRI	1.20 (1.17, 1.22)	< 0.001	1.33 (1.30, 1.37)	< 0.001	1.33 (1.30, 1.37)	< 0.001	1.25 (1.22, 1.28)	< 0.001
	BRI Quartile								
	Q1	1(Ref)		1(Ref)		1(Ref)		1(Ref)	
	Q2	1.32 (1.19, 1.46)	< 0.001	1.77 (1.57, 1.99)	< 0.001	1.80 (1.59, 2.04)	< 0.001	1.69 (1.33, 1.76)	< 0.001
	Q3	1.73 (1.54, 1.94)	< 0.001	2.77(2.42, 3.16)	< 0.001	2.78 (2.42, 3.20)	< 0.001	2.34 (1.82, 2.43)	< 0.001
	Q4	2.68 (2.38, 3.02)	< 0.001	5.07(4.41, 5.82)	< 0.001	5.11 (4.47, 5.85)	< 0.001	3.52 (2.74, 3.69)	< 0.001
	*P* for trend		< 0.001		< 0.001		< 0.001		< 0.001

*BRI* Body Roundness Index, *PIR* Poverty income ratio, *CVD* Cardiovascular disease, *DM* Diabetes mellitus, *DII* Dietary Inflammatory Index, *Asc* Ascorbic Acid, *OR* Odds ratio, *CI* Confidence interval, *β* Coefficient

Model 1: not adjusted

Model 2: adjusted for age, sex, and race

Model 3: adjusted for model 2, additionally adjusted for marital status, PIR, educational level, smoking status, alcohol intake, and physical activity

Model 4: adjusted for model 3, additionally adjusted for CVD, Hypertension, DM, Dietary fiber, DII, Zinc intake, Asc intake

### Subgroup and interaction analysis

**Fig. 3:**
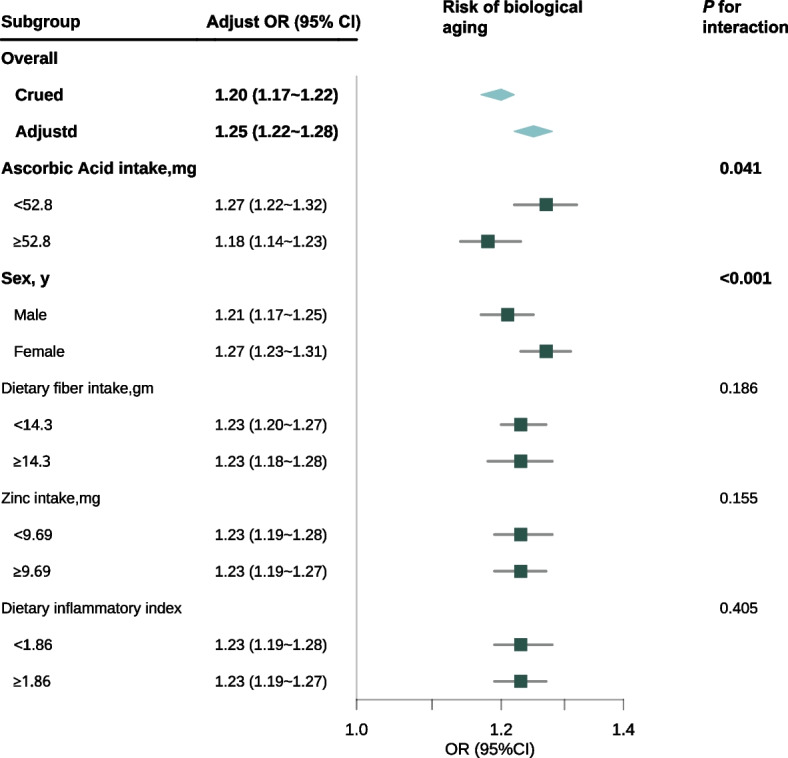
Subgroup analysis of biological aging risk was conducted using a multivariate weighted logistic regression mode OR: odds ratio, CI: confidence interval. This forest plot summarizes the adjusted OR with 95% CI for the risk of biological age acceleration across various subgroups, adjusted according to Model 4. The overall crude OR is 1.20 (95% CI: 1.17–1.22), and after adjustment, the OR is 1.25 (95% CI: 1.22–1.28). Interactions are significant for ascorbic acid intake (*P* for interaction = 0.041) and sex (*P* < 0.001), with females exhibiting an OR of 1.27 (95% CI: 1.23–1.31). No significant interactions are observed for dietary fiber intake, zinc intake, and dietary inflammatory index subgroups (*P* = 0.186, *P* = 0.155, *P* = 0.405, respectively). The diamonds denote the overall effect sizes, with horizontal Lines indicating 95% CIs

As illustrated in Fig. 3, the BRI consistently exhibited significant positive correlations with biological aging across all subgroups. Notably, interaction effects were identified between BRI and Asc intake (*P* = 0.041) as well as between BRI and sex (*P* < 0.001). However, in the subgroup with PA as the outcome, no interaction effect was found between BRI and Asc or sex (Supplemental Fig. 3)**.**

**Fig. 4:**
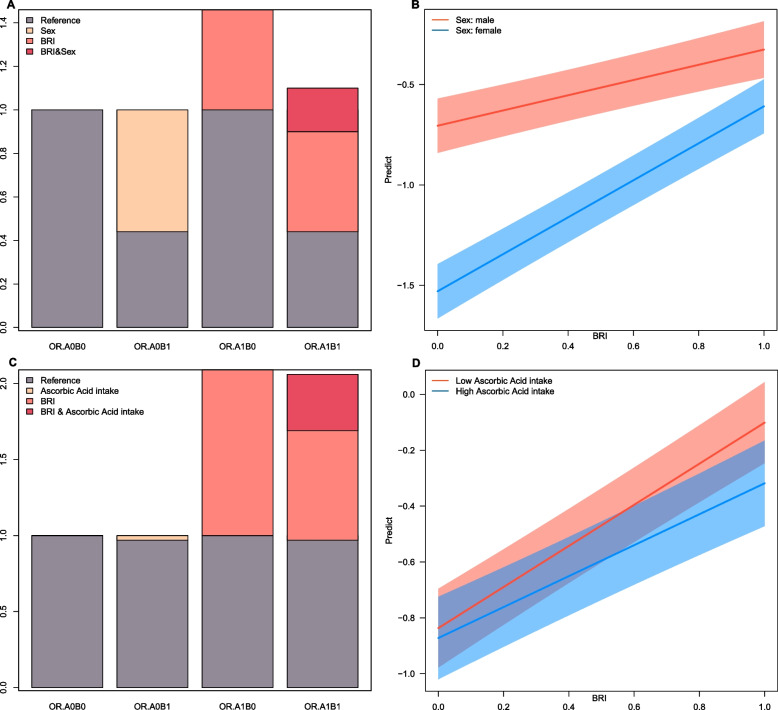
Interaction effects of BRI, Ascorbic Acid intake, and sex on biological aging This figure visualizes the interaction effects of BRI, ascorbic acid intake, and sex on biological aging, complementing the data pre-sented in Table 3. Panel A:Bar chart showing the odds ratios (OR) for biological aging across different groups: reference, sex, BRI, and the combined effect of BRI and sex. The combined effect (BRI & Sex) shows a higher OR compared to individual effects, indicating a significant interac-tion between BRI and sex on biological aging.Panel B:Line graph depicting the predicted biological aging outcomes for males and females across varying levels of BRI. The shaded areas represent 95% confidence intervals. The steeper slope for females suggests a stronger influence of BRI on biological aging in this group.Panel C:The bar chart, analogous to Panel A, concentrates on the interaction between ascorbic acid intake and the BRI. The combined effect of BRI and ascorbic acid intake demonstrates a lower OR compared to BRI alone, suggesting a potential mitigating association of ascorbic acid with BRI-related biological aging.Panel D:Line graph showing the predicted biological aging outcomes for individuals with low and high ascorbic acid intake across varying levels of BRI. Similar to Panel B, the shaded areas indicate 95% confidence intervals. The less steep slope for high ascorbic acid intake suggests a reduced impact of BRI on biological aging in this group

Significant multiplicative and additive interactions were detected between Asc intake and sex with respect to BA acceleration (Table 3, Fig. 4), with all* P*-values being < 0.05. On the multiplicative interaction scale, Asc intake was associated with a 17% reduction in biological aging (multiplicative scale: 0.83 [95% CI: 0.72–0.96], *P* = 0.01). Conversely, sex exhibited a significant positive association with biological aging (multiplicative scale: 1.72 [95% CI: 1.48–1.99], *P* < 0.001).

**Table 3:** BRI and the multiplicative and additive interactions of Ascorbic Acid intake and sex on biological aging

	Ascorbic Acid intake	Sex
Multiplicative interaction		
Multiplicative scale	0.83(0.72 ~ 0.96)	1.72(1.48 ~ 1.99)
*P*	0.01	< 0.001
Additive interaction		
RERI (95% CI)	−0.37 (−0.61 ~ −0.14)	0.20(0.05 ~ 0.36)
AP (95% CI)	−0.22 (−0.37 ~ −0.08)	0.18(0.04 ~ 0.32)
SI (95% CI)	0.65(0.5 ~ 0.84)	NA

*RERI* Relative excess risk due to interaction, *AP* Attributable proportion, *SI* Synergy index, *CI* Confidence interval

This table provides insights into the interaction effects of Ascorbic Acid intake and sex on biological aging as indicated by BRI. The multiplicative interaction results suggest that the impact of BRI on biological aging is modified by both Ascorbic Acid intake and sex. Specifically, the multiplicative scale for Ascorbic Acid intake, with a value less than 1, implies a reduced effect on biological aging when Ascorbic Acid intake is considered, indicating a potential protective role. Conversely, the multiplicative scale for sex, with a value greater than 1, suggests that biological aging is exacerbated in one sex compared to the other

The additive interaction measures provide further detail on how these factors influence biological aging independently of their main effects. The RERI values indicate that the joint effect of BRI, Ascorbic Acid intake, and sex on biological aging deviates from what would be expected if their effects were simply additive. The negative RERI for Ascorbic Acid intake suggests that the combined effect is less than additive, possibly due to a protective interaction, while the positive RERI for sex indicates a greater than additive effect, suggesting a synergistic interaction that enhances the impact of BRI on biological aging

The AP values quantify the proportion of the biological aging effect that is attributable to the interaction between BRI and the respective factors. The negative AP for Ascorbic Acid intake suggests that interactions reduce the risk of biological aging, while the positive AP for sex indicates that interactions increase this risk

According to the three measures of additive interaction, the RERI between Asc intake and BRI was calculated to be − 0.37 (95% CI: − 0.61 to − 0.14) for Asc intake, suggesting a protective interaction in this context. Conversely, the RERI for sex was 0.20 (95% CI: 0.05–0.36), indicating an elevated risk of BA acceleration. The AP due to interaction was − 0.22 (95% CI: − 0.37 to − 0.08) for Asc intake and 0.18 (95% CI: 0.04–0.32) for sex, implying that approximately 22% of the reduced risk in the Asc group and 18% of the increased risk in the sex group could be attributed to the interaction effect.

The SI for Asc intake was determined to be 0.65 (95% CI: 0.5–0.84), indicating that the combined effect of Asc intake and other factors was less than the sum of their individual effects, thereby demonstrating an antagonistic interaction. The SI for sex was not available (NA) in the analysis, which constrained our understanding of the potential synergistic effects associated with sex-related differences.

In conclusion, higher Asc consumption is associated with a reduced risk of biological aging in the context of the BRI, whereas increased BRI presents a more significant risk of biological aging in women than in men.

### Mediation analysis

**Fig. 5:**
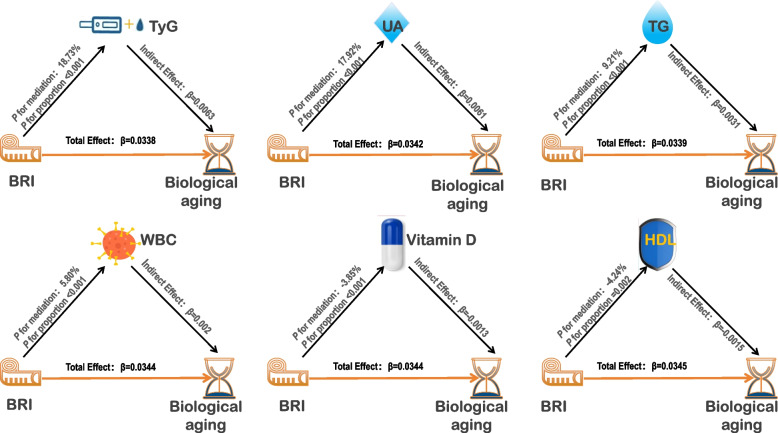
Mediation analysis of the relationship between BRI and biological aging BRI: Body Roundness Index, TG: triglyceride, WBC: white blood cell, VITD: vitamin D, UA: uric acid, HDL: High-Density Lipoprotein, TyG: triglyceride-glucose index. This diagram illustrates the mediation analysis of how BRI influences biological aging through various biomarkers, with adjustments made according to Model 4. The total effects of BRI on risk of biological aging are positive, indicating an overall accelerating effect of BRI on the aging process. The mediation proportions for each biomarker reflect the percentage of BRI's total effect that is mediated through that specific biomarker. Positive Indirect Effects: The biomarkers TyG, UA, TG, WBC, and Vitamin D exhibit positive indirect effects, indicating that BRI enhances biological aging both directly and indirectly through these mediators. The positive effects suggest that an increase in BRI levels is associated with higher levels of these biomarkers, which in turn are associated with an increased rate of biological aging. Negative Indirect Effects: The negative indirect effects observed for HDL and Vitamin D suggest that BRI may accelerate biological aging by reducing levels of these biomarkers. Specifically, the decrease in HDL and Vitamin D, which are generally associated with healthier biological aging, could contribute to an accelerated aging process. This implies that BRI's impact on biological aging is not solely direct but also operates through the modulation of these protective biomarkers

Mediation analysis indicated that, following adjustments in Model 4, several biomarkers exhibited significant mediating effects between BRI and biological aging (Fig. 5). UA demonstrated the most substantial positive mediating effect (17.92%, *P* < 0.001), followed by TyG (18.73%, *P* < 0.001), TG (9.21%, *P* < 0.001), and WBC count (5.80%, *P* < 0.001). Conversely, HDL (− 4.24%, *P* = 0.002) and VITD (− 3.85%, *P* < 0.001) exhibited significant negative mediating effects, indicating their protective roles. These results suggest that while IR, metabolic dysregulation, and inflammatory markers contribute to biological aging, HDL cholesterol and VITD may act as protective factors that moderate the relationship between BRI and biological aging.

### Sensitivity analyses

No significant alterations were observed in the multifactorial regression analysis after trimming extreme values of the BRI distribution (at the 0.5th and 99.5th percentiles; Supplemental Table 2). Furthermore, when the dependent variable was specified as PA, and biological aging was assessed through PA, a one-unit increment in the BRI was associated with a 0.97-year increase in PA (β = 0.97, 95% CI: 0.88–1.06, *P* < 0.001). This increase corresponded to a 31% heightened risk of biological aging (OR = 1.31, 95% CI: 1.27–1.35, *P* < 0.001). Among the quartile groups, individuals in the highest BRI category exhibited the most advanced PA and the greatest risk of biological aging (Supplemental Table 3). After directly deleting the missing covariates, the risk association between BRI and BA remained robust (Supplemental Table 4).

## Discussion

This large-scale study revealed an association between abdominal adiposity, as measured by the BRI, and accelerated biological aging, highlighting four key findings. First, a robust nonlinear BRI-aging relationship that persisted after extensive confounder adjustment was identified. Second, sex and Asc acid intake significantly modified this relationship, with females showing increased vulnerability, whereas higher Asc intake was associated with a protective relationship. Third, from a mechanistic perspective, the association between BRI and aging appears to be mediated by metabolic dysfunction and inflammation, while VITD and HDL demonstrated protective negative mediation effects (− 3.85% and − 4.24%, respectively). Fourth, threshold analyses revealed the critical inflection points at which the association between visceral adiposity and accelerated aging became significantly stronger. These findings highlight the complex interplay between abdominal obesity, sex-specific vulnerability, and modifiable dietary factors in biological aging, and the dual pathways of harmful and protective mechanisms.

While the observed positive, nonlinear association is consistent with the trends reported for BMI and the Cardiometabolic Index (CMI) [26–28], BRI offers superior specificity for visceral adiposity over a more general BMI. A more direct contrast is the Visceral Adiposity Index (VAI), for which complex, non-monotonic M-shaped relationships with aging biomarkers have been reported [20], creating challenges for clinical translation. In addition, there is a research gap in the waist-to-hip ratio (WHtR) in relation to comprehensive biological aging metrics. In contrast, a central contribution of the present analysis is that the BRI exhibits a straightforward, monotonically increasing association with both BA and PA. This clear dose–response relationship suggests that the BRI is a more pragmatic and interpretable tool for clinical risk stratification. This study further distinguishes itself by moving beyond simple associations to identify previously unexplored modifiers (sex and Asc intake) and providing a holistic mechanistic framework by integrating multiple metabolic and inflammatory pathways, addressing the fragmented nature of prior mediation analyses [29, 30].

A key finding of this study was the nonlinear association between the BRI and risk of biological aging, characterized by a distinct inflection point. This suggests a shift in the underlying biological response to increased visceral adiposity. Initially, a gentler slope may reflect a state in which homeostatic buffering systems (e.g., antioxidant reserves) actively mitigate metabolic stress. Thus, the inflection point could represent the biological threshold at which these compensatory mechanisms are overwhelmed. Beyond this point, the steep acceleration in aging likely reflects the activation of self-perpetuating vicious cycles involving inflammation, lipotoxicity, and insulin resistance, which exponentially amplify damage. This hypothesis provides a plausible explanation for the rapid decline observed at higher BRI levels and merits further investigation.

The observed sex differences, where females exhibit both higher BRI levels and a stronger association with accelerated aging, can be understood within the biological framework of menopausal transition. Before menopause, estrogen promotes metabolically protective subcutaneous fat distribution [31]. However, the mean cohort age of 47.5 years places many female participants within the perimenopausal window, a period characterized by declining estrogen that triggers a fundamental redistribution of adipose tissue towards the visceral compartment [11, 32, 33]. This shift directly contributed to a higher BRI. This biological model is consistent with our mediation results. The analysis identified metabolic dysregulation and inflammation as key pathways in the general association between the BRI and aging. Therefore, it is plausible that, as women accumulate more visceral fat during this life stage, their exposure to these specific aging-accelerating mechanisms intensifies, providing a compelling explanation for their heightened vulnerability. The observed protective effect of Asc in the context of high BRI is biologically plausible. As a potent antioxidant, Asc can directly neutralize reactive oxygen species (ROS) generated by dysfunctional visceral adipose tissue and is crucial for the regeneration of other key antioxidants, such as vitamin E. This capacity to mitigate oxidative stress is directly linked to its anti-inflammatory properties, as it can suppress the activation of pro-inflammatory signaling pathways such as NF-κB, which are chronically stimulated in obesity [34, 35]. These mechanisms are particularly relevant given that individuals with obesity often exhibit lower circulating Asc levels, possibly due to increased Asc consumption from heightened oxidative stress [36], suggesting that maintaining an adequate Asc status could be critical for counteracting the aging-related consequences of visceral adiposity.

Notably, significant interaction effects between sex and Asc were observed for BA, but not for PA. This divergence likely reflects fundamental conceptual differences between the two metrics. BA is constructed to measure **'**physiological deviation**'** from an age norm, making it sensitive to dynamic metabolic shifts [17, 18]. In contrast, PA was calibrated against the mortality risk, capturing more chronic cumulative damage [19]. This explains why the protective association of Asc, which may rapidly improve physiological states such as oxidative stress, was evident only in BA. Similarly, BA's sensitivity to hormonal metabolic fluctuations likely underlies its unique sex interaction, a dynamic effect potentially neutralized in the long-term mortality calibration of PA. This disparity underscores that BA and PA capture distinct aspects of aging, with BA reflecting immediate metabolic health, and PA representing an accumulated systemic risk.

Mediation analyses revealed three interrelated pathways that appeared to explain the association between the BRI and biological aging. First, metabolic dysfunction, indicated by elevated TyG index and TG levels, reflects systemic insulin resistance and lipotoxicity common to visceral obesity [24]. This dysregulation overwhelms mitochondrial beta-oxidation, leading to excessive production of ROS and accumulation of toxic lipid intermediates (e.g., ceramides) [37, 38], which impair cellular bioenergetics and promote senescence [39, 40]. Second, oxidative stress and systemic inflammation were evident due to increased UA and WBC levels [41]. Obese individuals demonstrate increased purine synthesis and augmented production of free fatty acids, which contribute to increased UA generation. Furthermore, renal excretion of UA is reduced, culminating in elevated systemic UA levels [42, 43]. This, in turn, creates a systemic pro-oxidant environment that directly inflicts oxidative damage to DNA (e.g., formation of 8-oxoG) and alters the function of epigenetically modifying enzymes, accelerating epigenetic dysregulation and genomic instability [7, 8]. Third, the depletion of protective factors, such as VITD (which can be sequestered in excess adipose tissue) and HDL (whose anti-inflammatory function is compromised by systemic inflammation), undermines antioxidant defenses and vascular homeostasis [44–46], further amplifying the risks associated with aging.

Clinical interpretation of these findings warrants further investigation. While the proportion of the effect mediated by any single factor was modest, its collective contribution was substantial. This highlights the fact that a large clinically relevant portion of obesity-driven aging occurs through a network of modifiable pathways. Therefore, each identified mediator, such as the TyG index and UA, serves as both a mechanistic link and an actionable therapeutic target. This framework provides a basis for moving beyond generic advice towards personalized strategies, where interventions can be tailored to a patient's specific metabolic or inflammatory profile to counteract accelerated aging more effectively.

### Study strengths and limitations

This study presents several methodological and translational advances in this field. From a methodological perspective, the implementation of the BRI, a metric specifically designed to quantify visceral adiposity, addresses the limitations of conventional anthropometric indices, such as BMI, in accurately capturing central fat distribution. The simultaneous application of the dual biological aging metrics BA and PA reduces the risk of outcome misclassification. Additionally, nonlinear modeling and mediation analyses provide a robust framework for dissecting the complex associations between adiposity and aging. Clinically, the identification of BRI-specific thresholds for accelerated aging facilitates risk stratification and enables early intervention in high-risk populations. This study also highlights the independent modifying roles of sex and Asc intake and identifies modifiable targets for mitigating adiposity-related aging. Furthermore, mediation pathways such as metabolic dysfunction and oxidative stress have revealed actionable mechanisms for therapeutic intervention. By integrating nationally representative data with sensitivity analyses, this study bridges mechanistic hypotheses with population-level evidence, supporting the potential use of adiposity metrics, such as the BRI, to guide targeted interventions against obesity-associated aging.

This study had some limitations. First, the cross-sectional design limits the ability to draw causal inferences regarding the temporal relationship between the BRI and biological aging. Nonetheless, the consistency of the findings across the various sensitivity analyses and mediation models underscores the robustness of the observed associations. Second, although rigorous covariate adjustments, nonlinear modeling, and subgroup analyses were employed to mitigate bias, the potential for residual confounding from unmeasured variables (e.g., dietary sodium intake and genetic predisposition) cannot be entirely ruled out. Third, although the use of sampling weights enhances national representativeness within the U.S. population, the generalizability of the findings to non-Western populations (such as Asian or European cohorts with distinct patterns of adiposity distribution) requires further validation. Furthermore, although missing data were addressed through multiple imputations, the possibility of selection bias due to non-random attrition remains a concern.

## Conclusions

An elevated BRI demonstrates a nonlinear association with accelerated biological aging—a relationship modified by sex and Asc intake. Clinically, these findings support the use of BRI thresholds for early risk stratification, prompting the targeted management of mediating pathways such as metabolic dysfunction and oxidative stress to potentially slow aging. This increased risk in females calls for sex-specific vigilance. Future research should move beyond these associations to validate these thresholds and test for causality through longitudinal studies. Our findings also provide a rationale for designing targeted trials to investigate the specific benefits of Asc supplementation in high-BRI populations.

## Supplementary Information

Supplementary Material 1. Calculation method of BA and PA

Supplementary Material 2. Definition and explanation of covariables

Supplementary Material 3. Sample Missing Data Overview

Supplementary Material 4. Linear regression model testing

Supplementary Material 5. Logistic regression model testing

Supplementary Material 6. Subgroup analysis of phenotypic aging risk

Supplementary Material 7. Sensitivity analysis

Supplementary Material 8. Sensitivity analysis

Supplementary Material 9. Sensitivity analysis

## Data Availability

The National Health and Nutrition Examination Survey (NHANES) data are publicly available at https://wwwn.cdc.gov/nchs/nhanes.

## Abbreviations

BA: Biological age
PA: Phenotypic age
BRI: Body Roundness Index
NHANES: National Health and Nutrition Examination Survey
Asc: Ascorbic Acid
TyG: Triglyceride-glucose
TG: Triglyceride
UA: Uric acid
WBC: White blood cell
HDL: High-density lipoprotein
CI: Confidence interval
CA: Chronological age
BMI: Body mass index
CVD: Cardiovascular disease
Ln-CRP: Ln-C-reactive protein
PIR: Poverty income ratio
IR: Insulin resistance
DM: Diabetes mellitus
VITD: Vitamin D
SE: Standard errors
RERI: Relative excess risk due to interaction
AP: Attributable proportion
SI: Synergy index
OR: Odds Ratio
DNA: Deoxyribonucleic acid
VAI: Visceral Adiposity Index
CMI: Cardiometabolic Index
