# Supplementary material for: Sex-stratified and ascorbic acid intake-modified associations between body roundness index and biological aging: a NHANES-based study on interactions and mediation
Source: Lipids Health Dis. 2025 Sep 19;24:281. doi: 10.1186/s12944-025-02708-1 (PMC12447621; doi:10.1186/s12944-025-02708-1)
Supplement: Supplementary file 1 — Supplementary Material 1. Calculation method of BA and PA [file 12944_2025_2708_MOESM1_ESM.docx]

**Calculation method of BA and PA**

Calculation method of biological Age


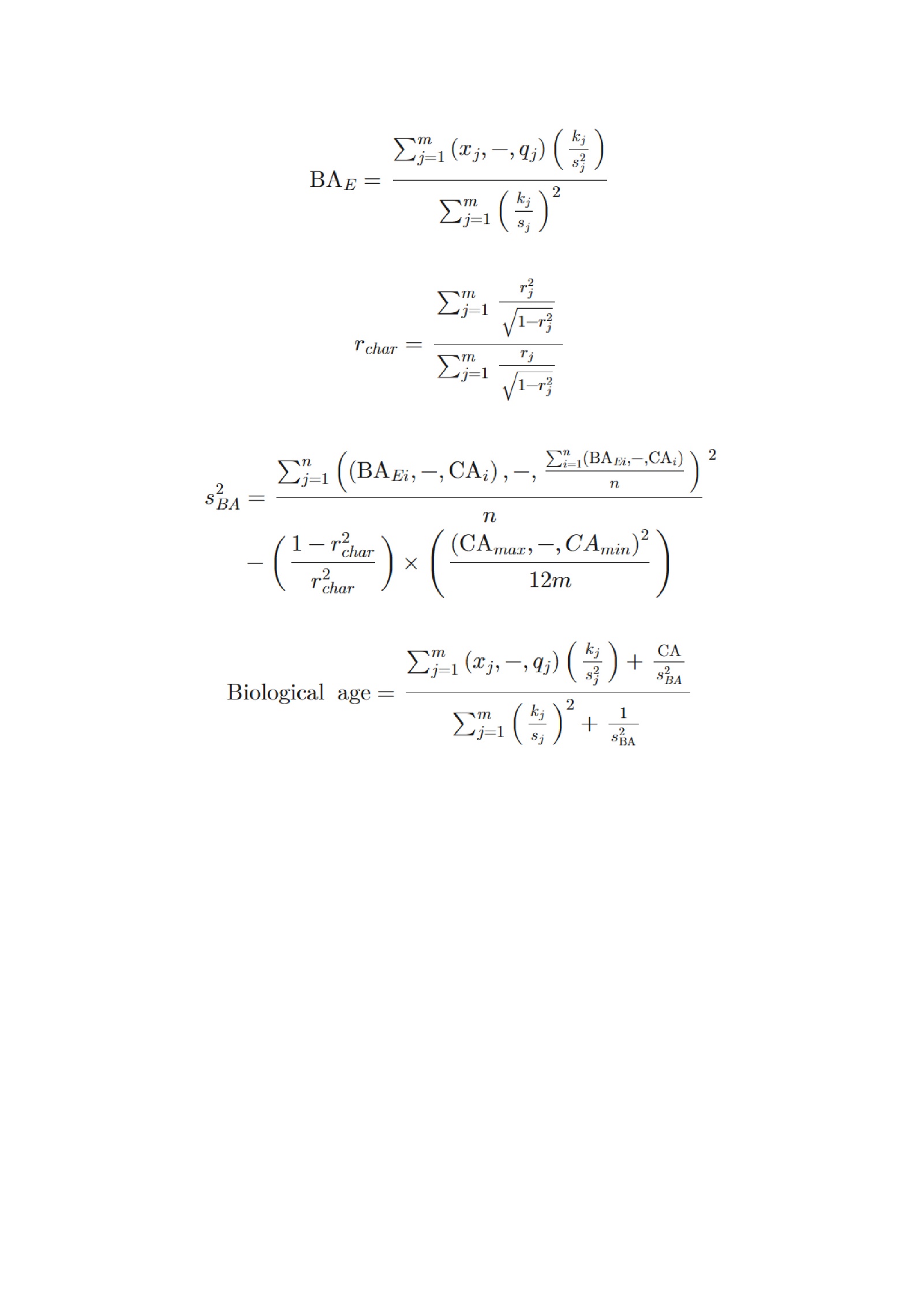


The values k, q, and s are the regression slope, intercept, and the root means squared error of a biomarker regressed on chronological age, respectively. The value rj2 represents the variance explained by regression of chronological age on biomarkers.

Calculation method of Phynotypic age

Then, based on the Gompertz distribution, these ten variables were included in a parametric proportional hazards model. According to this model, the 10-year mortality risk of the j-the individual was estimated based on the cumulative distribution function.

$${Mortality risk}_{j}=CDF\left( 120, x_{j} \right)=1-e^{x_{j}b(exp\left( 120\times\gamma\right)-1)/\gamma}$$

where xb represents the linear combination of biomarkers from the fitted model.

Next, the mortality risk score was converted into units of years using the following equation:

$$\mathrm{PhenoAge}_{j}=141.50+\frac{ln[-0.00553\times ln\left( 1-{Mortality risk}_{j} \right)]}{0.090165}$$

The final equations for calculating PhenoAge and PhenoAgeAccel in this study are as follows:

$$PhenoAge=141.50+\frac{\ln\left[ -0.00553\times\ln\left( 1-Mortality risk \right) \right]}{0.090165}$$

$$\mathrm{PhenoAgeAccel}=Phenotypic A\mathrm{ge}-Chronological Age$$

Where:

$$Mortality risk=1-exp(\frac{-1.51714\times exp(xb)}{0.0076927})$$

And:

$$xb=-19.907-0.0336\times albumin+0.0095\times Creatinine+0.1953\times Glucose+0.0954\times ln\left( \mathrm{CRP} \right)-0.0120\times Lymphocyte Percent+0.0268\times Mean Cell Volume+0.3306\times Red Cell Distribution Width+0.00188\times Alkaline Phosphatase+0.0554\times White Blood Cell Count+0.0804\times Chronological Age$$
