# Supplementary material for: Sex-stratified and ascorbic acid intake-modified associations between body roundness index and biological aging: a NHANES-based study on interactions and mediation
Source: Lipids Health Dis. 2025 Sep 19;24:281. doi: 10.1186/s12944-025-02708-1 (PMC12447621; doi:10.1186/s12944-025-02708-1)
Supplement: Supplementary file 2 — Supplementary Material 2. Definition and explanation of covariables [file 12944_2025_2708_MOESM2_ESM.docx]

**Definition and explanation of covariables**

Race: non-Hispanic White, non-Hispanic Black, mexican American, other Hispanic, other Race-Including Multi-Racial.

Marital status was categorized as: (1) Married/ Living with partner, and (2) Never married/Other: widowed, divorced, or separated individuals.

Education levels were classified as less than 9th grade, 9-11th Grade (Includes 12th grade with no diploma), high School Grad/GED or equivalent, some College or AA degree and college graduate or above

PIR was grouped into < 1.3, 1.3–3.5, and > 3.5.

Smoking status was categorized as never, former, and current

Alcohol intake categorized as never, former, and current.

Physical activity was assessed duration of activities per week.

CVD was defined as: A self-reported prior diagnosis of coronary heart disease, angina pectoris, stroke, myocardial infarction, or congestive heart failure, with the presence of any one of these conditions constituting a confirmed diagnosis.

Hypertension was diagnosed according to the following criteria, meeting any one of the following: 1. Self-reported prior physician diagnosis of hypertension 2. Current use of antihypertensive medications 3. Mean systolic blood pressure (SBP) ≥140 mmHg or mean diastolic blood pressure (DBP) ≥90 mmHg.

Diabetes mellitus was defined as meeting any one of the following criteria: 1. Self-reported physician-diagnosed diabetes 2. Glycated hemoglobin (HbA1c) ≥6.5% 3. Fasting plasma glucose (FPG) ≥7.0 mmol/L 4. Random plasma glucose (RPG) ≥11.1 mmol/L 5.2-hour oral glucose tolerance test (OGTT) glucose ≥11.1 mmol/L 6. Current use of antidiabetic medications or insulin.

Dietary fiber intake, zinc levels, and Ascorbic Acid concentrations were assessed using standardized dietary interview methodologies. Dietary intake information was utilized to calculate the type and amount of food and drink ingested in the 24 hours before the interview (from midnight to midnight) and to approximate calorie intake, nutrients, and other components from these sources.16 Each NHANES participant underwent 2 interviews recalling dietary intake over a 24-hour period. The primary interview was conducted face to face at a mobile examination center, and the next interview was set 3 to 10 days later, collected over phone. this study selected dietary fiber (g) on total nutrient intake on day 1, a 24-hour first meal recall interview personally collected by a mobile examination center and adjusted for individual weight factors. Sources of dietary fiber, including grains, vegetables, and fruits, are determined by sorting through relevant food categories.

Dietary Inflammatory Index (DII): A validated tool designed to assess the inflammatory potential of an individual's diet, calculated based on the effects of foods and dietary components on inflammatory biomarkers. The DII score is derived from six inflammatory markers: interleukin-1β (IL-1β), interleukin-4 (IL-4), interleukin-6 (IL-6), interleukin-10 (IL-10), tumor necrosis factor-α (TNF-α), and C-reactive protein (CRP). DII incorporates 45 dietary components, each contributing either pro- or anti-inflammatory effects to the overall inflammatory score. In the National Health and Nutrition Examination Survey (NHANES) database, 28 dietary components were utilized to calculate the DII score, including carbohydrates, protein, total fat, alcohol, fiber, cholesterol, saturated fat, monounsaturated fatty acids, polyunsaturated fatty acids, omega-3 (n-3) and omega-6 (n-6) fatty acids, niacin, vitamin A, vitamin B6, vitamin B12, vitamin C, vitamin D, vitamin E, thiamine, riboflavin, iron, magnesium, zinc, selenium, folate, β-carotene, caffeine, and total energy intake. Interpretation: Higher DII scores indicate a pro-inflammatory dietary pattern, whereas lower scores reflect an anti-inflammatory dietary profile.
