# Supplementary material for: Sex-stratified and ascorbic acid intake-modified associations between body roundness index and biological aging: a NHANES-based study on interactions and mediation
Source: Lipids Health Dis. 2025 Sep 19;24:281. doi: 10.1186/s12944-025-02708-1 (PMC12447621; doi:10.1186/s12944-025-02708-1)
Supplement: Supplementary file 3 — Supplementary Material 3. Sample Missing Data Overview [file 12944_2025_2708_MOESM3_ESM.docx]

Supplemental Table 1 Sample Missing Data Overview

| Variable | Valid Samples | Missing Samples | Missing Ratios, % |
| --- | --- | --- | --- |
| **Demographic** | | | |
| Age | 14337 | 0 | 0 |
| Sex | 14337 | 0 | 0 |
| Race | 14337 | 0 | 0 |
| Education level | 14325 | 12 | 0.0837 |
| Marital status | 14331 | 6 | 0.0418 |
| PIR | 13182 | 1155 | 8.0561 |
| **Lifestyle** | | | |
| Physical activity | 14337 | 0 | 0 |
| Smoking status | 14327 | 10 | 0.0697 |
| Alcohol intake | 13189 | 1148 | 8.0073 |
| **Health Status** | | | |
| CVD | 14336 | 1 | 0.007 |
| DM | 14337 | 0 | 0 |
| Hypertension | 14337 | 0 | 0 |
| **Dietary Intake** | | | |
| DII | 13876 | 461 | 3.2155 |
| Dietary fiber | 13876 | 461 | 3.2155 |
| Zinc intake | 13876 | 461 | 3.2155 |
| Ascorbic Acid intake | 13876 | 461 | 3.2155 |

PIR: poverty income ratio, CVD: Cardiovascular disease, DM: Diabetes mellitus, DII: Dietary Inflammatory Index,
