# Supplementary material for: Sex-stratified and ascorbic acid intake-modified associations between body roundness index and biological aging: a NHANES-based study on interactions and mediation
Source: Lipids Health Dis. 2025 Sep 19;24:281. doi: 10.1186/s12944-025-02708-1 (PMC12447621; doi:10.1186/s12944-025-02708-1)
Supplement: Supplementary file 4 — Supplementary Material 4. Linear regression model testing [file 12944_2025_2708_MOESM4_ESM.docx]

Supplemental Fig1 Linear regression model testing
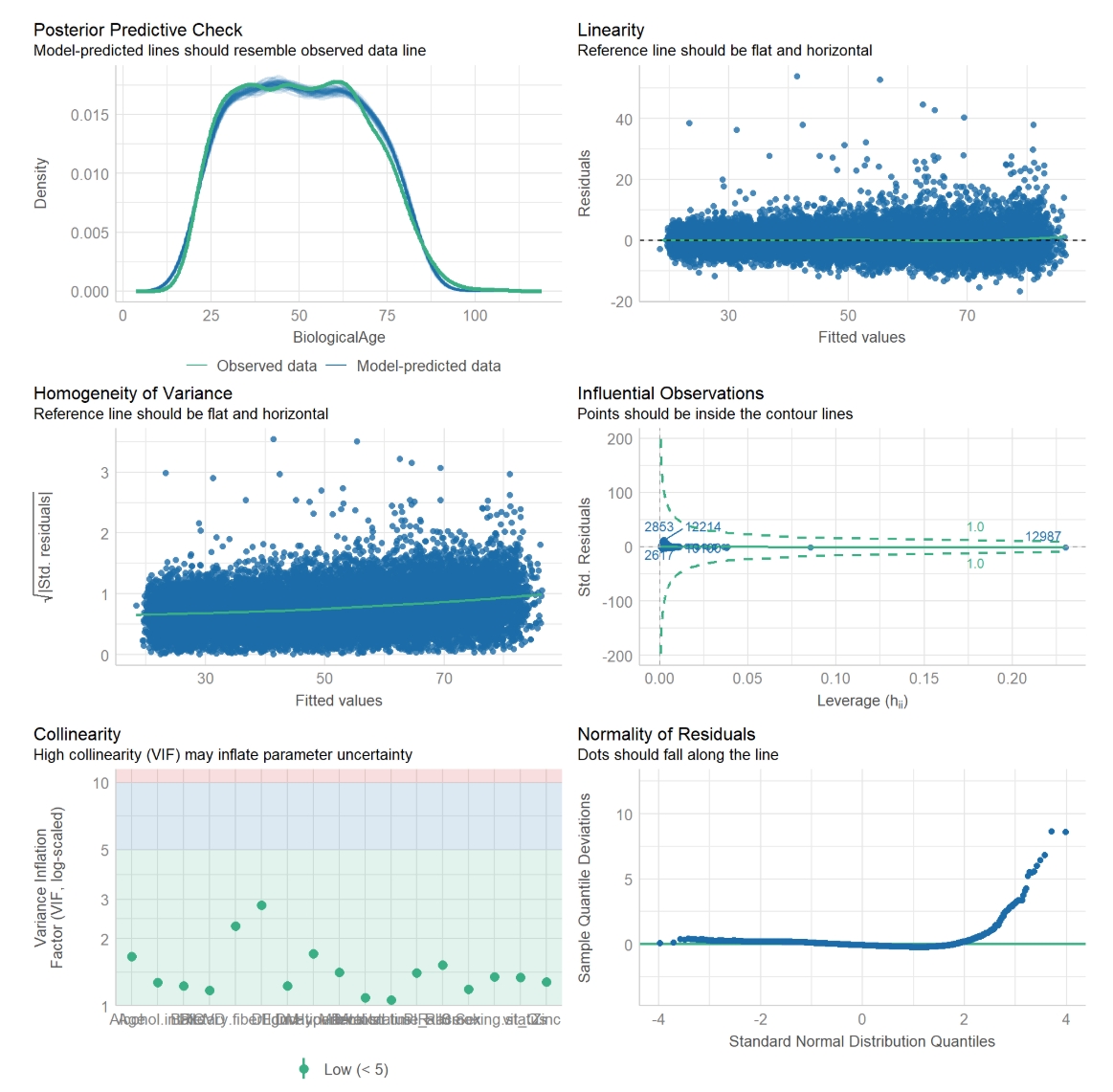


This figure presents a suite of diagnostic plots to assess the fit and assumptions of a regression model.

Posterior Predictive Check (Top Left): The density plot compares observed data (blue) with model-predicted data (green). The close resemblance of the two curves suggests that the model captures the distribution of the observed data well.

Linearity (Top Right): The residuals versus fitted values plot checks for linearity. The reference line should be flat and horizontal if the linearity assumption holds. The spread of points around the line indicates that the linearity assumption is met, with some deviations at the extremes.

Homogeneity of Variance (Middle Left): This plot assesses the homoscedasticity assumption, where residuals should have constant variance across all levels of fitted values. The flat and horizontal reference line suggests that the variance of residuals is consistent.

Influential Observations (Middle Right): The plot of standardized residuals against leverage identifies influential data points. Points within the contour lines are considered normal, while those outside may be influential outliers.

Collinearity (Bottom Left): The Variance Inflation Factor (VIF) plot assesses multicollinearity among predictors. VIF values below 5 (green dots) indicate low collinearity, while values above 10 (red area) suggest high collinearity, which could inflate parameter uncertainty.

Normality of Residuals (Bottom Right): The Q-Q plot checks the normality of residuals. Points should fall along the reference line if residuals are normally distributed. The slight deviation at the tails suggests some deviation from normality, but the overall pattern is acceptable.
