# Supplementary material for: Sex-stratified and ascorbic acid intake-modified associations between body roundness index and biological aging: a NHANES-based study on interactions and mediation
Source: Lipids Health Dis. 2025 Sep 19;24:281. doi: 10.1186/s12944-025-02708-1 (PMC12447621; doi:10.1186/s12944-025-02708-1)
Supplement: Supplementary file 5 — Supplementary Material 5. Logistic regression model testing [file 12944_2025_2708_MOESM5_ESM.docx]

Supplemental Fig.2 Logistic regression model testing
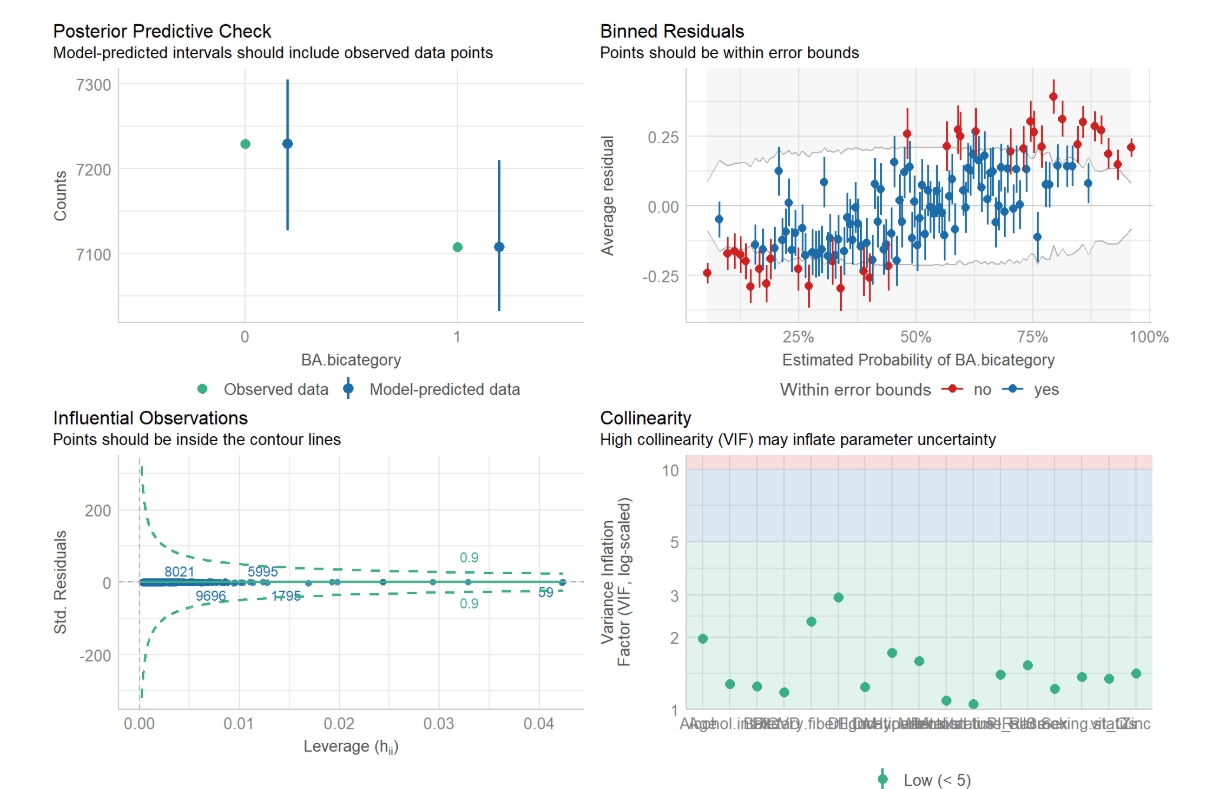
This figure provides a detailed examination of a regression model's fit and the potential issues of collinearity among predictors.

Posterior Predictive Check (Top Left):This plot compares observed data points (green dots) with model-predicted intervals (blue lines) across two BA bicategory groups. The overlap of observed data within the predicted intervals suggests that the model's predictions are consistent with the observed data.

Binned Residuals (Top Right):The plot displays average residuals across different estimated probabilities of BA bicategory. Points should fall within the error bounds (dashed lines) to indicate a good model fit. The presence of red points outside the bounds suggests some model predictions are less accurate.

Influential Observations (Bottom Left):This plot identifies influential data points by plotting standardized residuals against leverage. Points within the contour lines are considered normal, while those outside may significantly affect the model. The plot shows that most points are within the normal range, indicating minimal influence from outliers.

Collinearity (Bottom Right):The Variance Inflation Factor (VIF) plot assesses multicollinearity among predictors. VIF values below 5 (green dots) indicate low collinearity, which is desirable as high collinearity can inflate parameter uncertainty. The plot shows that all predictors have VIF values below the threshold, suggesting an acceptable level of collinearity.
