# Supplementary material for: Sex-stratified and ascorbic acid intake-modified associations between body roundness index and biological aging: a NHANES-based study on interactions and mediation
Source: Lipids Health Dis. 2025 Sep 19;24:281. doi: 10.1186/s12944-025-02708-1 (PMC12447621; doi:10.1186/s12944-025-02708-1)
Supplement: Supplementary file 6 — Supplementary Material 6. Subgroup analysis of phenotypic aging risk [file 12944_2025_2708_MOESM6_ESM.docx]

Supplemental Fig. 3 Subgroup analysis of phenotypic aging risk was conducted using a multivariate weighted logistic regression model

.
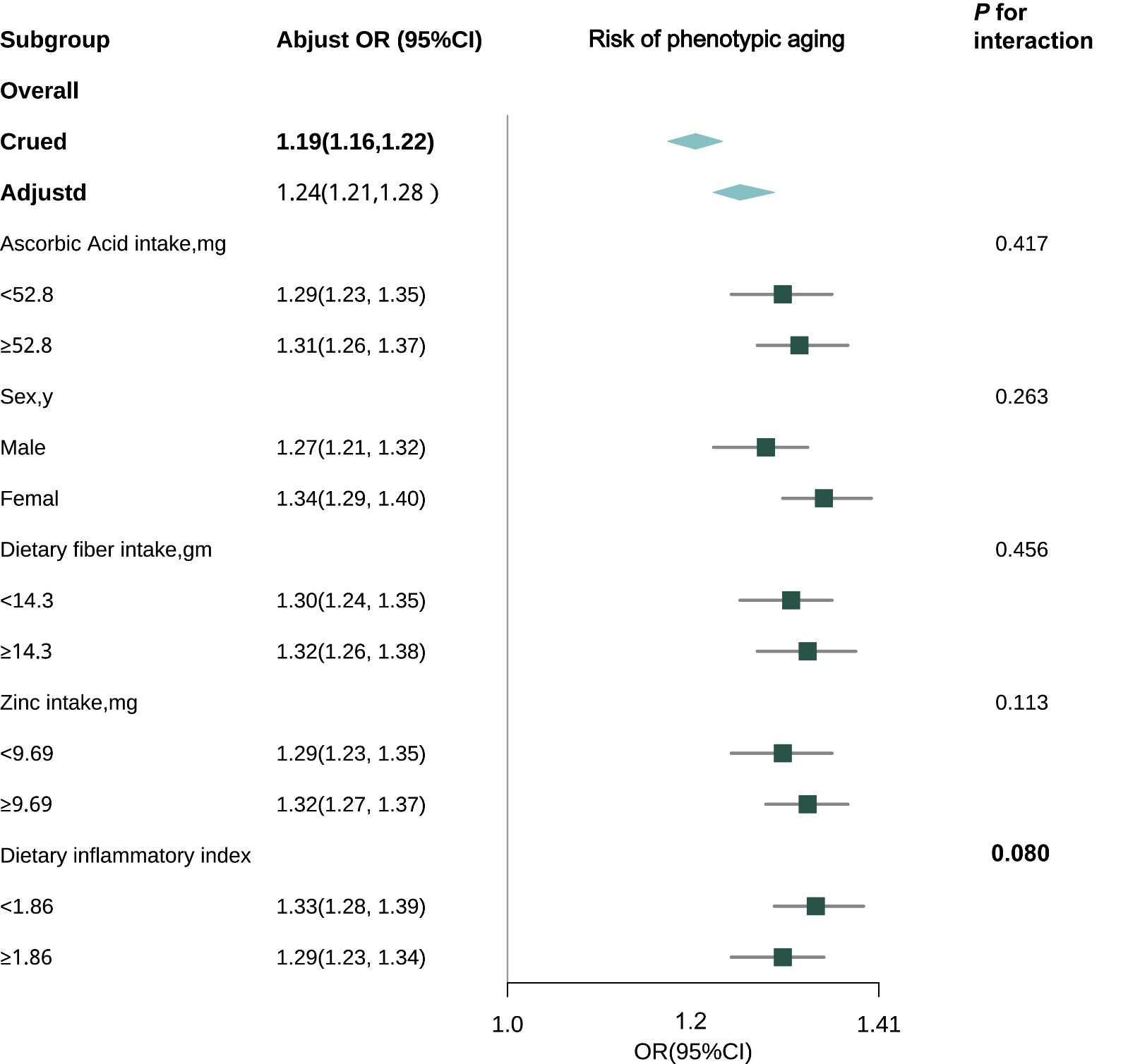


OR: odds ratio, CI: confidence interval

This forest plot summarizes the adjusted OR with 95% CI for the risk of biological age acceleration across various subgroups, adjusted according to Model 4. The overall crude OR is 1.20 (95% CI: 1.17-1.22), and after adjustment, the OR is 1.25 (95% CI: 1.22-1.28). No significant interactions are observed for ascorbic acid intake, sex, dietary fiber intake, zinc intake, and dietary inflammatory index subgroups (*P* = 0.417, *P* = 0.263, *P* = 0.456, *P* = 0.113, *P* = 0.080, respectively). The diamonds denote the overall effect sizes, with horizontal lines indicating 95% CIs.
