# Supplementary material for: Sex-stratified and ascorbic acid intake-modified associations between body roundness index and biological aging: a NHANES-based study on interactions and mediation
Source: Lipids Health Dis. 2025 Sep 19;24:281. doi: 10.1186/s12944-025-02708-1 (PMC12447621; doi:10.1186/s12944-025-02708-1)
Supplement: Supplementary file 7 — Supplementary Material 7. Sensitivity analysis [file 12944_2025_2708_MOESM7_ESM.docx]

| Table S2 Sensitivity analysis: Association of BRI with Biological Age and biological aging risk prediction after excluding data below the 0.5th percentile and above the 99.5th percentile | | | | | | | | | |
| --- | --- | --- | --- | --- | --- | --- | --- | --- | --- |
|  | Categories | **Model 1** | | **Model 2** | | **Model 3** | | **Model 4** | |
|  |  | β(95%CI) | *P-*value | β(95%CI) | *P-*value | β(95%CI) | *P-*value | β(95%CI) | *P-*value |
| **biological**  **age** | BRI | 2.30 (2.12,2.48) | <0.001 | 0.60 (0.55, 0.65) | <0.001 | 0.58(0.53,0.63) | <0.001 | 0.37 (0.33, 0.42) | <0.001 |
|  | BRI Quartile |  |  |  |  |  |  |  |  |
|  | Q1 | 0(Ref) |  | 0(Ref) |  | 0(Ref) |  | 0(Ref) |  |
|  | Q2 | 8.14(7.35,8.93) | <0.001 | 1.04 (0.82,1.26) | <0.001 | 1.05 (0.82,1.27) | <0.001 | 0.82 (0.60,1.04) | <0.001 |
|  | Q3 | 12.01 (11.07,12.95) | <0.001 | 1.96 (1.72,2.19) | <0.001 | 1.91 (1.68,2.15) | <0.001 | 1.35 (1.12,1.58) | <0.001 |
|  | Q4 | 14.04(13.06,15.01) | <0.001 | 3.25 (3.00,3.50) | <0.001 | 3.20 (2.95,3.45) | <0.001 | 2.03 (1.78,2.27) | <0.001 |
|  | P for trend |  | <0.001 |  | <0.001 |  | <0.001 |  | <0.001 |
|  |  |  |  |  |  |  |  |  |  |
| **biological**  **aging** |  | OR (95%CI) | *P-*value | OR (95%CI) | *P-*value | OR (95%CI) | *P-*value | OR (95%CI) | *P-*value |
|  | BRI | 1.20(1.17, 1.22) | <0.001 | 1.34 (1.30, 1.37) | <0.001 | 1.34 (1.30, 1.37) | <0.001 | 1.25 (1.21, 1.28) | <0.001 |
|  | BRI Quartile |  |  |  |  |  |  |  |  |
|  | Q1 | 0(Ref) |  | 0(Ref) |  | 0(Ref) |  | 0(Ref) |  |
|  | Q2 | 1.31 (1.18, 1.46) | <0.001 | 1.74(1.54, 1.96) | <0.001 | 1.77(1.56,2.01) | <0.001 | 1.66 (1.45,1.90) | <0.001 |
|  | Q3 | 1.71(1.52, 1.93) | <0.001 | 2.71(2.37, 3.10) | <0.001 | 2.73 (2.37,3.13) | <0.001 | 2.30 (1.99,2.65) | <0.001 |
|  | Q4 | 2.60 (2.30, 2.93) | <0.001 | 4.87(4.24, 5.59) | <0.001 | 4.90 (4.29,5.61) | <0.001 | 3.40 (2.93,3.93) | <0.001 |
|  | P for trend |  | <0.001 |  | <0.001 |  | <0.001 |  | <0.001 |

BRI: Body Roundness Index, PIR: poverty income ratio, CVD: Cardiovascular disease, DM: Diabetes mellitus, DII: Dietary Inflammatory Index, Asc: Ascorbic Acid, OR: odds ratio, CI: confidence interval, β: coefficient

Model 1: not adjusted

Model 2: adjusted for age, sex, and race

Model 3: adjusted for model 2, additionally adjusted for marital status, PIR, educational level, smoking status, alcohol intake, and physical activity

Model 4: adjusted for model 3, additionally adjusted for CVD, Hypertension, DM, Dietary fiber, DII, Zinc intake, Asc intak
