# Supplementary material for: Sex-stratified and ascorbic acid intake-modified associations between body roundness index and biological aging: a NHANES-based study on interactions and mediation
Source: Lipids Health Dis. 2025 Sep 19;24:281. doi: 10.1186/s12944-025-02708-1 (PMC12447621; doi:10.1186/s12944-025-02708-1)
Supplement: Supplementary file 8 — Supplementary Material 8. Sensitivity analysis [file 12944_2025_2708_MOESM8_ESM.docx]

Supplemental Table 3 Association of BRI with Phenotypic age and biological aging (defined by Phenotypic age) risk

|  | Categories | **Model 1** | | **Model 2** | | **Model 3** | | **Model 4** | |
| --- | --- | --- | --- | --- | --- | --- | --- | --- | --- |
| **Phenotypic**  **age** |  | β(95%CI) | *P-*value | β(95%CI) | *P-*value | β(95%CI) | *P-*value | β(95%CI) | *P-*value |
|  | BRI | 2.98(2.79,3.18) | <0.001 | 1.25(1.15,1.35) | <0.001 | 1.25(1.16,1.35) | <0.001 | 0.97(0.88,1.06) | <0.001 |
|  | BRI Quartile |  |  |  |  |  |  |  |  |
|  | Q1 | 0(Ref) |  | 0(Ref) |  | 0(Ref) |  | 0(Ref) |  |
|  | Q2 | 9.39(8.50,10.28) | <0.001 | 1.35(0.95,1.75) | <0.001 | 1.47(1.08,1.85) | <0.001 | 1.33(0.97,1.70) | <0.001 |
|  | Q3 | 14.41(13.34,15.48) | <0.001 | 3.01(2.56,3.47) | <0.001 | 3.11(2.66,3.55) | <0.001 | 2.48(2.05,2.91) | <0.001 |
|  | Q4 | 18.94(17.84,20.04) | <0.001 | 6.87(6.29,7.46) | <0.001 | 6.88(6.33,7.44) | <0.001 | 5.25(4.73,5.78) | <0.001 |
|  | P for trend |  | <0.001 |  | <0.001 |  | <0.001 |  | <0.001 |
|  | | | | | | | | | |
| **Biological**  **aging** |  | OR (95%CI) | *P-*value | OR (95%CI) | *P-*value | OR (95%CI) | *P-*value | OR (95%CI) | *P-*value |
|  | BRI | 1.35(1.32,1.38) | <0.001 | 1.35(1.32,1.39) | <0.001 | 1.37(1.34,1.41) | <0.001 | 1.31(1.27,1.35) | <0.001 |
|  | BRI Quartile |  |  |  |  |  |  |  |  |
|  | Q1 | 0(Ref) |  | 0(Ref) |  | 0(Ref) |  | 0(Ref) |  |
|  | Q2 | 1.55(1.35,1.79) | <0.001 | 1.44(1.24,1.67) | <0.001 | 1.52(1.31,1.77) | <0.001 | 1.47(1.27,1.71) | <0.001 |
|  | Q3 | 2.41(2.05,2.83) | <0.001 | 2.20(1.88,2.58) | <0.001 | 2.38(2.02,2.81) | <0.001 | 2.10(1.78,2.48) | <0.001 |
|  | Q4 | 5.28(4.58,6.09) | <0.001 | 5.12(4.37,6.00) | <0.001 | 5.66(4.82,6.66) | <0.001 | 4.31(3.65,5.09) | <0.001 |
|  | P for trend |  | <0.001 |  | <0.001 |  | <0.001 |  | <0.001 |

BRI: Body Roundness Index, PIR: poverty income ratio, CVD: Cardiovascular disease, DM: Diabetes mellitus, DII: Dietary Inflammatory Index, Asc: Ascorbic Acid, OR: odds ratio, CI: confidence interval, β: coefficient

Model 1: not adjusted

Model 2: adjusted for age, sex, and race

Model 3: adjusted for model 2, additionally adjusted for marital status, PIR, educational level, smoking status, alcohol intake, and physical activity

Model 4: adjusted for model 3, additionally adjusted for CVD, Hypertension, DM, Dietary fiber, DII, Zinc intake, Asc intake
