# Supplementary material for: Sex-stratified and ascorbic acid intake-modified associations between body roundness index and biological aging: a NHANES-based study on interactions and mediation
Source: Lipids Health Dis. 2025 Sep 19;24:281. doi: 10.1186/s12944-025-02708-1 (PMC12447621; doi:10.1186/s12944-025-02708-1)
Supplement: Supplementary file 9 — Supplementary Material 9. Sensitivity analysis [file 12944_2025_2708_MOESM9_ESM.docx]

| Supplemental Table 4 Association of BRI with Biological Age and Biological Aging Risk (Missing values are directly excluded) | | | | | | | | | |
| --- | --- | --- | --- | --- | --- | --- | --- | --- | --- |
|  | Categories | **Model 1** | | **Model 2** | | **Model 3** | | **Model 4** | |
|  |  | β(95%CI) | *P-*value | β(95%CI) | *P-*value | β(95%CI) | *P-*value | β(95%CI) | *P-*value |
| **biological**  **age** | BRI | 2.10 (1.92, 2.28) | <0.001 | 0.58 (0.53, 0.63) | <0.001 | 0.57 (0.52, 0.62) | <0.001 | 0.37 (0.32, 0.41) | <0.001 |
|  | BRI Quartile |  |  |  |  |  |  |  |  |
|  | Q1 | 0(Ref) |  | 0(Ref) |  | 0(Ref) |  | 0(Ref) |  |
|  | Q2 | 8.12 (7.35, 8.90) | <0.001 | 1.11 (0.87, 1.34) | <0.001 | 1.09 (0.85, 1.33) | <0.001 | 0.85 (0.61, 1.10) | <0.001 |
|  | Q3 | 12.03 (11.04, 13.03) | <0.001 | 2.07 (1.81, 2.33) | <0.001 | 2.01 (1.75, 2.28) | <0.001 | 1.43 (1.18, 1.68) | <0.001 |
|  | Q4 | 13.79 (12.85, 14.74) | <0.001 | 3.34 (3.06, 3.62) | <0.001 | 3.29 (3.02, 3.57) | <0.001 | 2.08 (1.80, 2.36) | <0.001 |
|  | *P* for trend |  | <0.001 |  | <0.001 |  | <0.001 |  | <0.001 |
|  |  |  |  |  |  |  |  |  |  |
| **biological**  **aging** |  | OR (95%CI) | *P-*value | OR (95%CI) | *P-*value | OR (95%CI) | *P-*value | OR (95%CI) | *P-*value |
|  | BRI | 1.19 (1.16, 1.22) | <0.001 | 1.33 (1.29, 1.37) | <0.001 | 1.33 (1.29, 1.37) | <0.001 | 1.24 (1.21, 1.28) | <0.001 |
|  | BRI Quartile |  |  |  |  |  |  |  |  |
|  | Q1 | 1(Ref) |  | 1(Ref) |  | 1(Ref) |  | 1(Ref) |  |
|  | Q2 | 1.34 (1.18, 1.51) | <0.001 | 1.81 (1.58, 2.07) | <0.001 | 1.83 (1.59, 2.10) | <0.001 | 1.70 (1.46, 1.98) | <0.001 |
|  | Q3 | 1.78 (1.57, 2.03) | <0.001 | 2.92(2.52, 3.38) | <0.001 | 2.91 (2.51, 3.38) | <0.001 | 2.42 (2.08, 2.81) | <0.001 |
|  | Q4 | 2.68 (2.33, 3.07) | <0.001 | 5.16(4.41, 6.05) | <0.001 | 5.20 (4.45, 6.07) | <0.001 | 3.51 (2.96, 4.15) | <0.001 |
|  | *P* for trend |  | <0.001 |  | <0.001 |  | <0.001 |  | <0.001 |

BRI: Body Roundness Index, PIR: poverty income ratio, CVD: Cardiovascular disease, DM: Diabetes mellitus, DII: Dietary Inflammatory Index, Asc: Ascorbic Acid: OR: odds ratio, CI: confidence interval, β: coefficient

Model 1: not adjusted

Model 2: adjusted for age, sex, and race

Model 3: adjusted for model 2, additionally adjusted for marital status, PIR, educational level, smoking status, alcohol intake, and physical activity

Model 4: adjusted for model 3, additionally adjusted for CVD, Hypertension, DM, Dietary fiber, DII, Zinc intake, Asc intake
